# The Effect of Aqueous Extract of Cinnamon on the Metabolome of* Plasmodium falciparum* Using ^1^HNMR Spectroscopy

**DOI:** 10.1155/2016/3174841

**Published:** 2016-01-20

**Authors:** Shirin Parvazi, Sedigheh Sadeghi, Mehri Azadi, Maryam Mohammadi, Mohammad Arjmand, Farideh Vahabi, Somye Sadeghzadeh, Zahra Zamani

**Affiliations:** ^1^Young Researchers and Elite Club, Islamic Azad University, Damghan Branch, Damghan, Semnan 3671639998, Iran; ^2^Biochemistry Department, Pasteur Institute of Iran, Pasteur Avenue, Tehran 1316943551, Iran

## Abstract

Malaria is responsible for estimated 584,000 deaths in 2013. Researchers are working on new drugs and medicinal herbs due to drug resistance that is a major problem facing them; the search is on for new medicinal herbs. Cinnamon is the bark of a tree with reported antiparasitic effects. Metabonomics is the simultaneous study of all the metabolites in biological fluids, cells, and tissues detected by high throughput technology. It was decided to determine the mechanism of the effect of aqueous extract of cinnamon on the metabolome of* Plasmodium falciparum in vitro* using ^1^HNMR spectroscopy. Prepared aqueous extract of cinnamon was added to a culture of* Plasmodium falciparum* 3D7 and its 50% inhibitory concentration determined, and, after collection, their metabolites were extracted and ^1^HNMR spectroscopy by NOESY method was done. The spectra were analyzed by chemometric methods. The differentiating metabolites were identified using Human Metabolome Database and the metabolic cycles identified by Metaboanalyst. 50% inhibitory concentration of cinnamon on* Plasmodium falciparum* was 1.25 mg/mL with *p* < 0.001. The metabolites were identified as succinic acid, glutathione, L-aspartic acid, beta-alanine, and 2-methylbutyryl glycine. The main metabolic cycles detected were alanine and aspartame and glutamate pathway and pantothenate and coenzyme A biosynthesis and lysine biosynthesis and glutathione metabolism, which are all important as drug targets.

## 1. Introduction

Malaria is one of the major infectious diseases particularly in tropical countries. It is caused by the protozoan parasite of genus* Plasmodium* and transmitted by the female* Anopheles* mosquito [1].

So far, only 5 of the 170 species of this parasite have been found which are the cause of disease in humans. They consist of* Plasmodium falciparum*,* Plasmodium knowlesi*,* Plasmodium ovale*,* Plasmodium vivax*, and* Plasmodium malariae* [2].

*Plasmodium falciparum* is the most dangerous of all and can even lead to death. The latest released statistics by December 2014 showed 198 million cases of malaria in 2013 comprised of estimated 584,000 deaths. Malaria mortality rates have fallen by 47% globally since 2000 and by 54% in the WHO African Region [3].

Since the 17th century, the bark of the* Cinchona* tree which was the source of quinine had been the first effective western treatment for* malaria* [4]. However, chloroquine replaced it from the 1940s, although quinine is still used under certain circumstances [5]. The resistance against antimalaria drugs is a drawback of standard drugs like chloroquine, sulphadoxine-pyrimethamine, and even artemisinin. The search for new herbal drugs is of prime importance [6].

Cinnamon consists of cinnamaldehyde compounds, volatile oils, tannins, mucilage, limonene, and safrole that possesses antibacterial, antiseptic, antiviral, and antifungal properties [7]. Senhaji et al. in 2005 tested different extracts of cinnamon like the aqueous, hexane, methanol, and ethanol on gram positive and negative bacteria as well as yeast,* Leishmania*, and* Toxoplasma* with positive results [8].

More recently, Nkanwen and colleagues in 2013 tested the bark of cinnamon for antiplasmodial activity and found that it had an inhibitory effect on* Plasmodium falciparum* enoyl-ACP reductase enzyme [9].

Metabonomics is the recent omics that studies simultaneously all the metabolites and small molecules in biological fluids, cells, and tissues. It uses high throughput technology like ^1^H Nuclear Magnetic Resonance (^1^HNMR) and Liquid Chromatography Mass Spectrometry (LC-MS). It plays the most important role in direct observation of the physiological status of an organism or the cell and is a faster and more affordable way of testing drugs and their mechanism of action [10].

Earlier studies have reported antiplasmodial effect of cinnamon extract and its result on one of its enzymes. It was decided to study the metabolome of* Plasmodium falciparum* after exposure to cinnamon extract by ^1^HNMR spectroscopy.

## 2. Materials and Methods

### 2.1. Preparation of Cinnamon Extract

*Cinnamomum cassia* obtained from Mumbai, India, were ground into a fine powder. 50 grams of cinnamon powder was dissolved in 500 mL of distilled water and boiled for 3 hours and then filtered through a gauze. The obtained extract was concentrated into an oily extract using a rotary machine and then lyophilized to 12.48 grams of cinnamon powder.

### 2.2. *Plasmodium falciparum* Culture

Strains of 3D7 provided by the late Dr. Walliker were cultured using the method of Trager and Jensen. Briefly, the parasites were cultured in 7 mL RPMI 1640 medium with 5% serum, 10% hematocrit, hypoxanthine, and gentamicin (complete medium) in 75 mL flasks. The medium was changed every 48 h and flasks were incubated at 37°C with 5% CO_2_, 5% O_2_, and 90% N_2_ [11].

Large-scale cultivation of* Plasmodium falciparum* for metabonomics was carried out by the method modified by Radfar et al. with 24 h changes of 75 mL of complete medium enriched with 5% albumax and 0.5% hematocrit. Daily monitoring of the culture assisted in increasing its parasitemia to 60% and the IC50 dose of cinnamon extract. Cultures grown without drugs were used as negative controls. After 48 hours, they were harvested by centrifugation at 800 g for 5 min [12].

### 2.3. IC50 Determination

Parasites reaching 5% were then diluted to reduce the parasitemia to 0.5%, and the haematocrit was adjusted to 1.5%. This suspension was then added (100 *μ*L per well) to microplates predosed with 90 *μ*L of different concentration of cinnamon or artemisinin and incubated for 48 hours at 37°C in mixed gas of 5% CO_2_, 5% O_2_, and 90% N_2_; after that thin smears were prepared from each well, stained with giemsa stain for determination of percentage of parasitemia and IC50 detected by microscopy [13].

### 2.4. Isolation of Parasites

Parasites were isolated by adding 40 times the volume of 0.02% saponin in phosphate buffered saline (PBS) on ice for 30 min and then centrifuged at 4,000 g for 20 min at 4°C. The cells were washed three times with 1XPBS and pellet collected by centrifugation in the above conditions and final centrifugation was carried out at 14,000 g for 5 min at 4°C; the cells were counted in a hemocytometer and stored at −20°C [14].

### 2.5. Preparation of Parasite Extract

The samples containing parasites sonicated in a sonifier (Soniprep 150) at 9 KHz for 5 min in pulse were then centrifuged at 10000 g for 10 min, and the pellet dissolved in 200 *μ*L of 1.8 mM cold perchloric acid and pH adjusted by addition of 5.4 M KOH to 6.8 and kept on ice for 60 min to precipitate the acid. The parasite extract was then centrifuged for 10 min at 10,000 g and the pH once again adjusted to 6.8 and lyophilized [14].

### 2.6. Preparation of Sample for ^1^HNMR

1 mL of D_2_O and 0.01% TSP was added to the lyophilized powder and spectroscopy was performed using 2-dimensional NOESY (Nuclear Overhauser Spectroscopy) conditions [15].

### 2.7. Computational Analysis

The spectra from ^1^HNMR were Fourier transformed by Mestrec software. To obtain regression values, the variables of the signal intensities and chemical shifts were integrated and were inserted into the Excel file. Normal intensities were used for further analysis with MATLAB.

### 2.8. Partial Linear Square (PLS)

PLS is a supervised method to obtain a model using regression in multivariate techniques via linear combination of original variables in which *X* is the normal intensities from the ^1^HNMR spectra and the *Y* matrix comprises 0 for cinnamon treated and 1 for controls. Orthogonal signal correction (OSC) filters removed noise from the spectrum; only one factor was removed and PLS was applied after OSC [16].

### 2.9. Identification of Metabolites

Metabolites corresponding to these resonances were then identified using chemical shift assignments of spectra of differentiating metabolites of sera based on comparison with chemical shifts of metabolites in Human Metabolite Database Data Bank (HMDB) (http://www.hmdb.ca/metabolites) [17] and in other published data. Analysis of metabolite cycles was carried out using Metaboanalyst software (http://www.metaboanalyst.ca) [18].

## 3. Results

**Figure 1:**
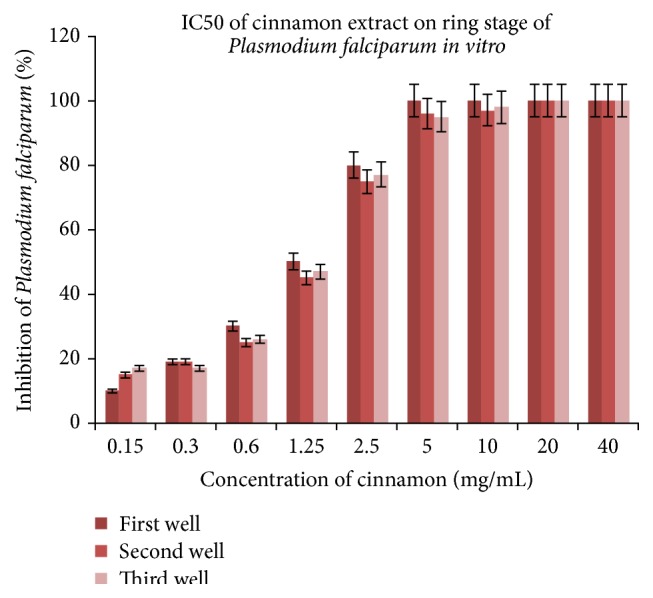
Determination of IC50 of cinnamon on* Plasmodium falciparum in vitro*.

**Figure 2:**
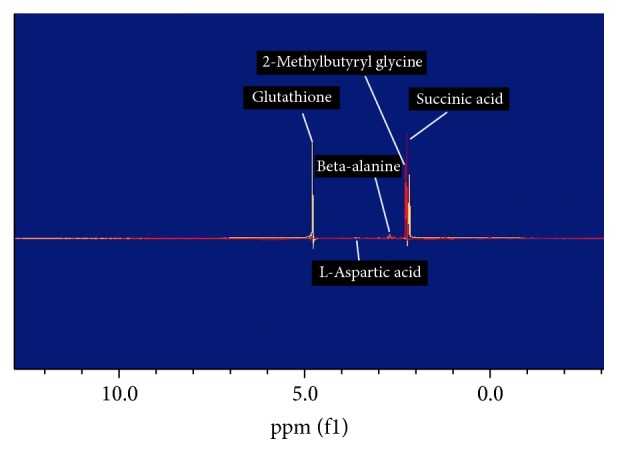
Superimposed spectra of cinnamon treated* Plasmodium falciparum*.

**Figure 3:**
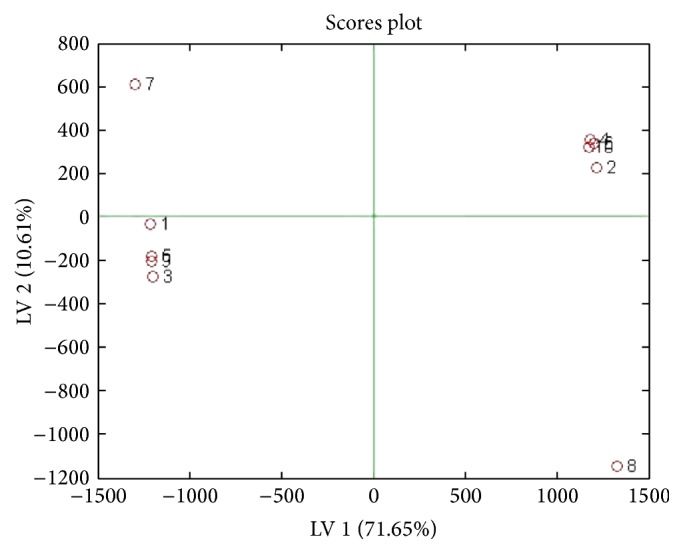
Score plot of OSC-PLS of samples depicting complete separation of the two groups of samples. Odd numbers show cinnamon treated samples and even numbers show controls.

**Figure 4:**
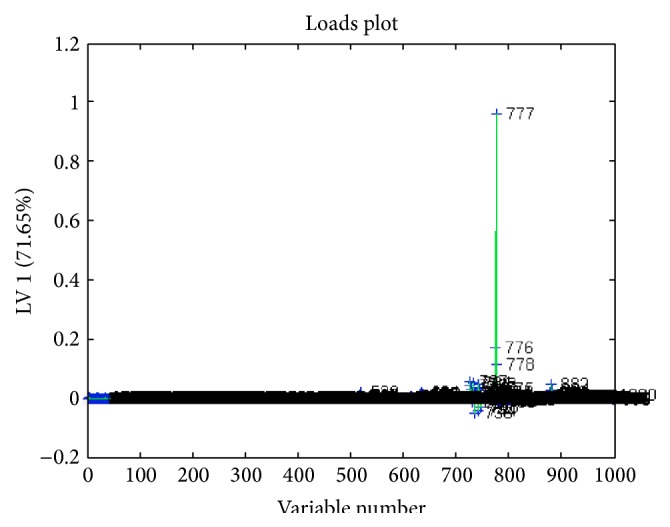
Loading plot demonstrating the differentiating metabolites between the two groups.

**Figure 5:**
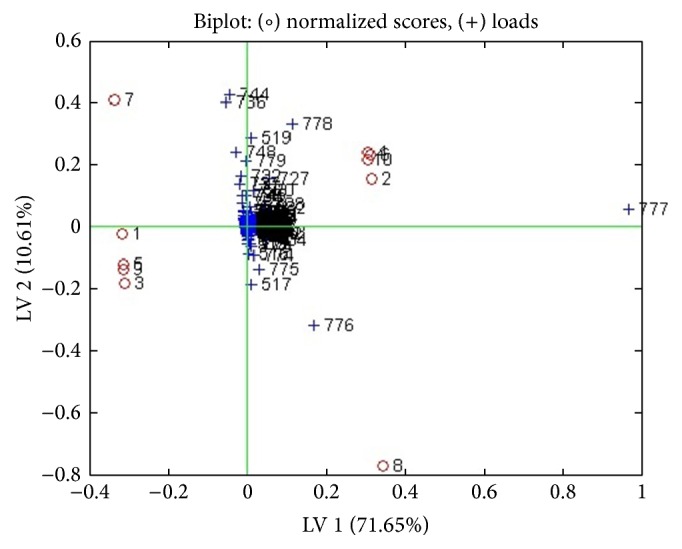
Biplot showing separation of the samples (numbered circles) and differentiating metabolites (crosses). Outlier crosses show the most significant changed metabolites in* Plasmodium*.

**Figure 6:**
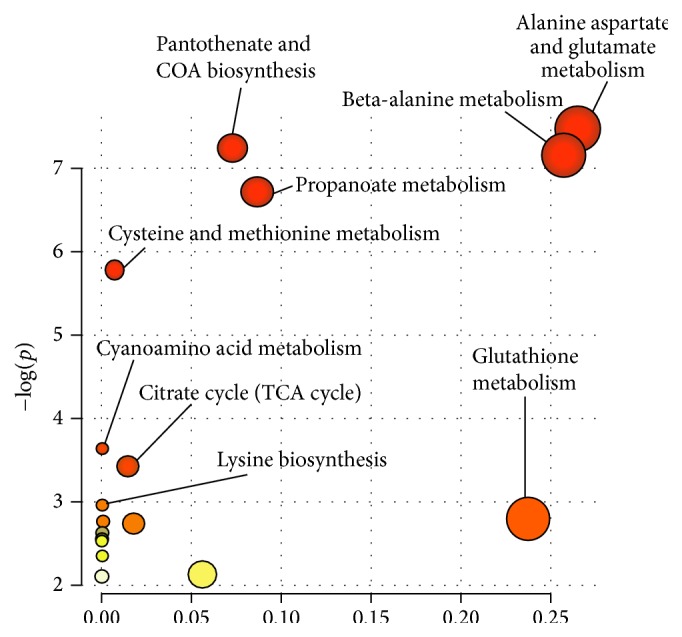
Pathway analysis showing all matched pathways according to *p* values from pathway enrichment analysis and pathway impact values from pathway topology analysis.

The lyophilized cinnamon was redissolved in RPMI medium and tested on 5%* Plasmodium falciparum* and IC50 of 1.25 mg/mL was obtained with significance *p* < 0.001 (Figure 1). Parasite extract obtained from large-scale cultivation of* Plasmodium falciparum* was analyzed by ^1^HNMR. The spectra of the cinnamon treated* Pasmodium falciparum* and controls were superimposed in Figure 2. The chemical shifts (ppm) of the spectra were converted into figures and then analyzed using OSC-PLS in MATLAB.  Figure 3 shows complete separation of the two groups of samples.  Figure 4 shows differentiating metabolites between the two groups.  Figure 5 depicts the biplot showing both the score plot and loading plot. The outliers indicate the most significant differentiating metabolites which are detected from their numbers. The metabolites were identified from their chemical shifts using HMDB (Table 1). The metabolites were entered into the Metaboanalyst database and differentiating metabolic cycles were recognized (Figure 6).

## 4. Discussion

Cinnamon has IC50 of 1.25 mg/mL on* Plasmodium falciparum in vitro* with IC50 of 1.25 mg/mL obtained with significance *p* < 0.001. The altered metabolites comprise succinic acid, glutathione, L-aspartic acid, beta-alanine, and 2-methylbutyryl glycine (Table 1). The most significant biochemical pathways which have changed are discussed below (Figure 6).

The alanine, aspartame, and glutamate pathway which is one of the amino acid cycles is the first one to be affected. L-Aspartic and succinic acids are the metabolites which take part in it. There are very early reports about the ability of the parasite to fix carbon dioxide and then synthesize alanine, aspartame, and glutamate. But amino acid uptake by the parasite from the infected erythrocytes is confirmed [19]. When culturing* Plasmodium falciparum in vitro* seven amino acids have to be supplied exogenously; they are isoleucine, methionine, cysteine, glutamate, glutamine, proline, and tyrosine [20]. Proteases act on amino acids especially aspartic proteases, as probable drug targets till a few years ago [21]. It is seen that cinnamon affects the parasite's amino acid biosynthesis essential for its survival.

The second most important pathway affected is pantothenate and coenzyme A biosynthesis associated directly with the tricarboxylic acid (TCA) cycle. Pantothenate is a precursor of the fundamental enzyme cofactor coenzyme A (CoA). Reports have shown that infected human erythrocytes exhibit higher coenzyme A biosynthesis than uninfected ones. It is important for growth of the intraerythrocytic stage of human malaria. Human erythrocytes are capable of complete synthesis of pantothenate and CoA unlike rat and avian erythrocytes [22]. Cinnamon affects this cycle which is imperative in TCA and requires the carriage of carbons within the cell and entry of pyruvate to the tricarboxylic acid (TCA) cycle. It was thought that* Plasmodium falciparum* utilized glucose in an unconventional manner, but further work by metabolic approaches has proved that both asexual and sexual blood stages utilize a conventional TCA cycle to catabolize glucose and glutamine [23, 24].

The next important cycle to be affected is lysine biosynthesis which has been reported in the parasite,* Plasmodium falciparum*. Lysine is generally implicated in posttranslational modifications and is also involved in diverse cellular functions. High throughput technology has identified 421 acetylation sites in 230 proteins in the human malaria parasite* Plasmodium falciparum* during its active proliferation in erythrocytes. Lysine-acetylated proteins are distributed in the nucleus, cytoplasm, mitochondrion, and apicoplast [25]. There are also reports of histone lysine methylation, regulated by methyltransferases and demethylases, which play an important role in chromatin structure and gene expression in a wide range of eukaryotic organisms. The SET-domain protein methyltransferase superfamily includes all but one of the proteins known to methylate histones on lysine. Histone methylation is important in the regulation of chromatin and gene expression. Bioinformatic analysis has shown that nine SET-domain-containing genes were found in the malaria parasite* Plasmodium falciparum* and its sibling species. Most SET-domain genes and histone methyl-lysine marks displayed dynamic changes during the parasite asexual erythrocytic cycle, suggesting that they constitute an important epigenetic mechanism of gene regulation in malaria parasites [26]. They are considered as recent targets for designing of antimalarial drugs and cinnamon extract affects them [27].

Glutathione metabolism is the last and most important cycle which has shown a change in the metabolome of* Plasmodium falciparum* to cinnamon. It is reported that* Plasmodium falciparum* employs a complex thioredoxin and glutathione system based on the thioredoxin reductase/thioredoxin and glutathione reductase/glutathione couples.* Plasmodium falciparum* thioredoxin reductase reduces thioredoxin and a range of low molecular weight compounds, while glutathione reductase is highly specific for its substrate glutathione disulfide. Since* Plasmodium* spp. lack catalase and a classical glutathione peroxidase, their redox balance depends on a complex set of five peroxiredoxins differentially located in the cytosol, apicoplast, mitochondria, and nucleus with partially overlapping substrate preferences. Moreover,* P. falciparum* employs a set of members belonging to the thioredoxin superfamily such as three thioredoxins, two thioredoxin-like proteins, a dithiol and three monocysteine glutaredoxins, and a redox-active plasmoredoxin with largely redundant functions. It is seen that glutathione metabolism is disturbed by the cinnamon extract.

It can be concluded that cinnamon has an inhibitory effect on* Plasmodium falciparum in vitro* with IC50 of 1.25 mg/mL with significance of *p* < 0.001. The altered metabolites are succinic acid, glutathione, L-aspartic acid, beta-alanine, and 2-methylbutyryl glycine and the main metabolic cycles affected were alanine, aspartate, and glutamate pathway, pantothenate and coenzyme A biosynthesis, lysine biosynthesis, and glutathione metabolism, all of which are important as drug targets.

## Figures and Tables

**Table 1:** Differentiating metabolites identified by their chemical shifts using HMDB.

Differentiating metabolites	HMDB
Succinic acid	HMDB00254
Glutathione	HMDB00121
L-Aspartic acid	HMDB00191
Beta-alanine	HMDB00056
2-Methylbutyryl glycine	HMDB00339
