# Corrigendum to “The Effect of Aqueous Extract of Cinnamon on the Metabolome of* Plasmodium falciparum* Using ^1^HNMR Spectroscopy”

**DOI:** 10.1155/2016/9275636

**Published:** 2016-06-28

**Authors:** Shirin Parvaz, Sedigheh Sadeghi, Mehri Azadi, Maryam Mohammadi, Mohammad Arjmand, Farideh Vahabi, Somye Sadeghzadeh, Zahra Zamani

**Affiliations:** ^1^Young Researchers and Elite Club, Islamic Azad University, Damghan Branch, Damghan, Semnan 3671639998, Iran; ^2^Biochemistry Department, Pasteur Institute of Iran, Pasteur Avenue, Tehran 1316943551, Iran

In the article titled “The Effect of Aqueous Extract of Cinnamon on the Metabolome of* Plasmodium falciparum* Using ^1^HNMR Spectroscopy” [1], the name of the first author was incorrectly written as Shirin Parvazi, while the correct name is Shirin Parvaz. The correct author list is shown above.
